# EFhd2 co-aggregates with monomeric and filamentous tau *in vitro*

**DOI:** 10.3389/fnins.2024.1373410

**Published:** 2024-05-03

**Authors:** Ahlam S. Soliman, Andrew Umstead, Jared Lamp, Irving E. Vega

**Affiliations:** ^1^Department of Translational Neuroscience, College of Human Medicine, Michigan State University, Grand Rapids, MI, United States; ^2^Neuroscience Program, Michigan State University, East Lansing, MI, United States; ^3^Integrated Mass Spectrometry Unit, College of Human Medicine, Michigan State University, Grand Rapids, MI, United States; ^4^Department of Neurology, University of Michigan, Ann Arbor, MI, United States; ^5^Michigan Alzheimer's Disease Research Center, University of Michigan, Ann Arbor, MI, United States

**Keywords:** EFhd2, tau, neurofibrillary tangles, protein aggregation, Alzheimer’s disease, tauopathies

## Abstract

Tauopathies are characterized by the abnormal buildup of tau protein, with early oligomeric forms associated with neurodegeneration and the later neurofibrillary tangles possibly conferring neuroprotection. The molecular mechanisms governing the formation of these tau species are unclear. Lately, there has been an increased focus on examining the interactions between tau and other proteins, along with their influence on the aggregation of tau. Our previous work revealed EFhd2’s association with pathological tau in animal models and tauopathy brains. Herein, we examined the impact of EFhd2 on monomeric and filamentous tau *in vitro*. The results demonstrated that EFhd2 incubation with monomeric full length human tau (hTau40) formed amorphous aggregates, where both EFhd2 and hTau40 colocalized. Moreover, EFhd2 is entangled with arachidonic acid (ARA)-induced filamentous hTau40. Furthermore, EFhd2-induced aggregation with monomeric and filamentous hTau40 is EFhd2 concentration dependent. Using sandwich ELISA assays, we assessed the reactivity of TOC1 and Alz50—two conformation-specific tau antibodies—to EFhd2-hTau40 aggregates (in absence and presence of ARA). No TOC1 signal was detected in EFhd2 aggregates with monomeric hTau40 whereas EFhd2 aggregates with hTau in the presence of ARA showed a higher signal compared to hTau40 filaments. In contrast, EFhd2 aggregates with both monomeric and filamentous hTau40 reduced Alz50 reactivity. Taken together, our results illustrate for the first time that EFhd2, a tau-associated protein, interacts with monomeric and filamentous hTau40 to form large aggregates that are starkly different from tau oligomers and filaments. Given these findings and previous research, we hypothesize that EFhd2 may play a role in the formation of tau aggregates. Nevertheless, further *in vivo* studies are imperative to test this hypothesis.

## Introduction

The quest to treat tauopathies has fueled a drive to understand the formation of pathological tau aggregates and their effects on neurodegenerative conditions. Tau proteins, known for their dynamic and flexible nature, interact with various cellular components, which makes them central players in many cellular processes (Uversky, 2015; Wang and Mandelkow, 2016; Chung et al., 2021; Mueller et al., 2021). Tau function is regulated by post-translational modifications and interactions with other proteins (Uversky, 2015; Wang and Mandelkow, 2016; Chung et al., 2021). The dynamic structure of tau, coupled with these modifications, is believed to trigger the development of pathological tau variants (Nachman et al., 2020). While neurofibrillary tangles (NFTs) were long considered the primary culprits of neurodegeneration, research has uncovered a disconnect between tangle accumulation and neuronal loss (Morsch et al., 1999; Wittmann et al., 2001; Kuret et al., 2005; SantaCruz et al., 2005; Spires et al., 2006; Sydow et al., 2011; Cowan and Mudher, 2013; Kuchibhotla et al., 2014). In tandem with this evolving insight, oligomeric tau forms have emerged as potential instigators of neurodegeneration, and their accumulation correlates with cognitive decline (Berger et al., 2007; Brunden et al., 2008; Spires-Jones et al., 2009; Kayed, 2010; Cowan and Mudher, 2013). Still, identifying the toxic tau species remains a focal point of research, as does understanding the mechanisms behind tau aggregation.

Tau activity heavily relies on its interactions with other proteins, and these interactions shed light on tau pathology (Uversky, 2015; Kavanagh et al., 2022). For instance, T-cell intracellular antigen 1 (TIA1) is an RNA-binding protein that nucleates RNA stress granules. TIA1 has been demonstrated to interact with tau and induce its aberrant folding and neurodegeneration. TIA1 knockdown rescued tau-mediated neurotoxicity (Vanderweyde et al., 2016). Thus, reducing TIA1 can mitigate tau oligomerization at the expense of increasing tangle accumulation and enhance neuronal survival (Apicco et al., 2018). Along similar lines, the interaction between chaperones and tau has been largely investigated by several research groups using *in vitro* and *in vivo* models (Miyata et al., 2011). Recently a small heat shock protein 22 (HSP22) has been shown to prevent heparin-induced tau aggregation and reduced oligomeric tau build up (Darling et al., 2021).

Previously, we identified EFhd2 as a novel protein associated with tau in the brains of JNPL3 mouse model and postmortem Alzheimer’s disease (AD) and frontotemporal lobar degeneration (FTLD) cases (Vega et al., 2008). EFhd2 is a calcium-binding protein expressed in various organs, including the central nervous system (Vega et al., 2008; Purohit et al., 2014; Vega, 2016). We showed that EFhd2 protein abundance is increased in AD brain, co-purified with tau in the sarkosyl-insoluble fraction, and colocalized with pathological tau in AD brains, particularly in the somatodendritic compartment (Ferrer-Acosta et al., 2013b). Using immunogold electron microscopy, we found that EFhd2 and tau colocalized in filamentous structures, indicating that EFhd2 co-aggregates with tau (Ferrer-Acosta et al., 2013b).

In our quest to further understand EFhd2’s interactions with tau, we demonstrated that EFhd2 induces a conformational change in tau. When EFhd2 was incubated with the microtubule binding region of 3R tau (K19), without external aggregation inducers such as heparin or arachidonic acid, an elevated Thioflavin S signal (ThS) was observed (Vega et al., 2018). We also showed that EFhd2’s coiled-coil (C-C) domain facilitates its direct protein–protein interaction with tau *in vitro* (Ferrer-Acosta et al., 2013a). Furthermore, we reported that EFhd2 affects tau’s liquid–liquid phase separation by promoting the formation of solid-like structures (Vega et al., 2019). These findings led us to hypothesize that EFhd2 plays a role in the biogenesis of pathological tau aggregation in AD. This hypothesis is supported by the elevated EFhd2 levels in AD brains. An independent study confirmed that *Efhd2* is a target gene of miR-126a-3p, a microRNA that is downregulated in AD (Pichler et al., 2017; Xue et al., 2022). The downregulation of EFhd2 by miR-126-3p was shown to enhance memory consolidation and rescue cognitive deficits in a transgenic mouse model of AD (Xue et al., 2022).

Herein, we tested the hypothesis that EFhd2 associates with tau and promotes its aggregation. We examined the effect that recombinant human EFhd2 (hEFhd2) has on monomeric and filamentous full length human tau (hTau40) *in vitro*. Electron microscopy analysis revealed that hEFhd2 interacted with monomeric hTau40 forming amorphous aggregates. Furthermore, when hEFhd2 was combined with *in vitro*-formed hTau40 filaments/oligomers, hEFhd2 and hTau40 filaments became entangled forming unique larger aggregates. Immunogold labeling confirmed the colocalization of hEFhd2 and hTau40 in these distinct structures. Furthermore, these aggregates showed differential reactivity to conformation-specific tau antibodies; TOC1 and Alz50. Indeed, our study is the first to report the capacity of a protein, EFhd2, to interact with tau *in vitro* promoting the formation of higher order structures where they colocalize. Hence, this study provides the basis for further *in vivo* experiments to explore how EFhd2 modulates the biogenesis of tau aggregates in various tauopathies.

## Materials and methods

### Recombinant protein production

#### Recombinant EFhd2 and GST

The wild type human EFhd2 genes tagged with N-terminal 6x histidine or GST was subcloned into chemically competent BL21 (DE3) *E. coli* (New England Biolabs, cat# C2527H) cells as detailed in (Vega et al., 2008, 2019). Protein purification protocol was adapted from (Vega et al., 2019). Briefly, bacteria were inoculated from the frozen glycerol stock in a starter culture of 50 mL LB/Ampicillin (50 μg/mL) overnight at 37°C with constant shaking at 200 rpm. The next day, the saturated starter culture was diluted to 300 mL LB/Ampicillin (50 μg/mL) to 0.2–0.3 OD600 nm and incubated at 37°C with constant shaking at 250 rpm. When the culture reached 0.5–0.7 OD600 nm, a final concentration of 0.5 mM IPTG was added to induce protein expression. The culture was incubated for 1.5 h (hEFhd2) or 1 h (GST) at 37°C with constant shaking at 250 rpm. Immediately after the induction, OD600 was recorded to verify bacterial growth. The culture was centrifuged at 30,000 g for 10 min at 4°C. Then, the bacterial pellet was frozen at −80°C for 20 min. Afterwards, the pellet was resuspended in 10 mL lysis buffer (1X PBS with 5 mM imidazole). With respect to GST, the pellet was resuspended in 1X PBS. The resuspended pellet was further sonicated by using Misonix XL-2000 set at 4 on ice four times 20 s pulses, which was shortly followed by centrifugation at 33,000 g for 10 min at 4°C. The supernatant was rapidly separated and incubated with 1 mL of pre-equilibrated fresh HIS-select Nickel resin (Sigma, cat#H0537-25ML) or fresh GST Sepharose beads (GE Healthcare, cat# 17–0756-01) overnight at 4°C with constant rotation. Next, the beads were allowed to settle by gravity on ice, and the supernatant was discarded. The beads were then resuspended in 1 mL Lysis buffer (or 1X PBS for GST) and carefully transferred to 10 mL column (Biorad, cat#731–1,550). The Lysis buffer was allowed to flow through. As soon as the lysis buffer reaches the top of the beads bed, fresh 10 mL lysis buffer (or 1X PBS) was added to wash the beads. The recombinant protein was eluted with 500 μL of 1x PBS containing 250 mM Imidazole (pH 8.0) (or 50 mM glutathione for GST). Two fractions were collected for each protein and checked on SDS-PAGE. Then, the fractions were pooled and underwent three buffer exchange cycles with 1X PBS using centricon spin filters 3 kDa cutoff at 18,000 x g for 10 min (Sigma, cat# UFC500324). Protein concentration was determined by Pierce Rapid Gold BCA protein assay kit (Thermo Scientific, cat#A53225). To prevent hEFhd2 spontaneous self-aggregation, the final concentration was quickly brought to 2–2.5 μg/μL. For simplicity, GST final concentration was 2.3 μg/μL. The purified proteins were digested with trypsin and subjected to tandem mass spectrometry to identify potential post-translational modifications and bacterial protein contaminants (Umstead et al., 2020).

#### Recombinant tau protein

Recombinant tau production and purification protocol was adapted from (Combs et al., 2017) with modifications. DNA plasmid of full-length human tau (hTau40) with C-terminal 6x histidine tag (PT7CHT40) was transformed to chemically competent BL21 *E. coli* (New England Biolabs, cat#C2527H) cells. In particular, 10 ng of DNA was added to bacterial cells and mixed by gentle swirling followed by incubation on ice for 30 min. Then, the bacteria were exposed to heat shock at 42°C for exactly 30 s immediately followed by incubation on ice for 10 min. Then, transformed cells were allowed to recover by growing in 250 μL of antibiotic-free S.O.C medium at 37°C with constant shaking at 225 rpm for 1 h. Afterwards, cells were plated on prewarmed LB agar/Ampicillin (100 μg/mL) and incubated overnight at 37°C in inverted position. The next day, a single colony was picked and inoculated in a pre-culture of 50 mL LB/Ampicillin (100 μg/mL), which was incubated overnight at 30°C with constant shaking at 100 rpm. The saturated pre-culture was diluted to 300 mL with LB/Ampicillin (100 μg/mL) to <0.1 OD600 nm and incubated at 37°C with constant shaking at 250 rpm. When the culture reached 0.8–1 OD600 nm, a final concentration of 1 mM IPTG was added to induce protein expression. The culture was incubated for 2 h at 37°C with constant shaking at 250 rpm. The culture was centrifuged at 8000 g for 10 min at 4°C. The pellet was resuspended/ washed in 40 mL ice-cold STE buffer (0.1 M NaCl +10 mM Tris Base +1 mM EDTA, PH = 8.0). The cell suspension was carefully transferred to a pre-weighed tube and centrifuged at 8000 x g for 10 min at 4°C. The pellet weight was recorded. Afterwards, the pellet was resuspended in 5x volumes of ice-cold lysis D buffer (0.5 M NaCl +10 mM tris base +5 mM imidazole, pH = 8.0) containing 1x protease inhibitors cocktail (Thermo Fisher, cat# 78437) and 1 mM PMSF (Sigma, cat# 78830-5G). Then the pellet was sonicated by using Misonix XL-2000 set at 4 on ice four times with 20 s pulses. To avoid protein degradation, the protease inhibitors and PMSF were added after sonication in addition to 0.1% Brij 35 (Thermo Scientific, cat# 20150). The resulting lysate was boiled at 99°C for 15 min. This step is important to eliminate bacterial heat shock proteins purified with tau. The boiled lysate was centrifuged at 16,000 g for 10 min at 4°C. The supernatant was carefully transferred to a new tube wherein (1x) protease inhibitors and (1 mM) PMSF were added. Subsequently, the supernatant was incubated with 1 mL of pre-equilibrated fresh HIS-select Nickel resin (Sigma, cat#H0537-25ML) overnight at 4°C with constant rotation. The beads were allowed to settle by gravity on ice, and the supernatant was carefully removed and discarded. The beads were gently resuspended in 1 mL of Lysis buffer (1X PBS + 5 mM imidazole) and rapidly transferred to a 10 mL column (Biorad, cat#731–1,550). As the Lysis buffer reached the top of the beads bed, 10 mL of lysis buffer were added at once to wash the beads. Recombinant tau protein was eluted with 500 μL of 1X PBS containing 250 mM Imidazole (pH = 8.0). Two elution fractions were collected and checked on SDS-PAGE. The two fractions were then pooled and underwent three buffer exchange cycles with tau storage buffer (70 mM Tris, pH 7.4, 75 mM NaCl) using centricon spin filters 3 kDa cutoff at 18,000 x g for 10 min/ cycle at 4°C (Sigma, cat# UFC500324). Protein concentration was determined by Pierce Rapid Gold BCA protein assay kit (ThermoScientific, cat#A53225). The final concentration was brought to 2.5–5 μg/μL. Finally, DTT was added for a final concentration of 1 mM to impede the formation of disulfide bonds. The purified proteins were digested with trypsin and subjected to tandem mass spectrometry to identify potential post-translational modifications and bacterial protein contaminants (Umstead et al., 2020).

## *In vitro* tau polymerization and filament formation

Arachidonic acid (ARA) is a well-known polyanion molecule capable of inducing tau filaments *in vitro*. Being a free fatty acid, ARA promotes tau aggregation above critical micelle concentration due to the negative charge on the lipid surface, which acts as a nucleating factor for tau fibrillization (Wilson and Binder, 1997; Chirita et al., 2003). It is important to note that ARA promotes tau aggregation at 2 μM, which is similar to the physiological level of tau. Herein, we followed the standard protocol of ARA-induced tau aggregation (Combs et al., 2017) by adding 2 μM of recombinant protein (hTau40, hEFhd2, or GST) to the polymerization buffer (5 mM DTT + 100 mM NaCl +10 mM HEPEs +0.1 mM EDTA). The protein is mixed by gentle swirling and tapping. The final concentration of 75 μM ARA (Cayman, cat# 900100.1) was added carefully and mixed by gentle swirling to avoid air bubbles, which might change the aggregation dynamics. A working solution of 2 mM ARA in 100% ethanol is prepared immediately before use. Then, it is discarded due to the oxidation of ARA. After adding ARA to the polymerization reaction, the tubes are tightly wrapped using parafilm to minimize the evaporation that will impact the final concentration. Unless otherwise stated, the polymerization was allowed to proceed overnight (16–18 h) at room temperature. Reactions that do not include ARA, equivalent volume of 100% ethanol is added to the polymerization buffer-protein mixture. Equimolar concentrations (2 μM) were added to the polymerization buffer when recombinant proteins were incubated together. Moreover, to examine the effect of reducing EFhd2 concentration on tau aggregation, 2, 1 or 0.5 μM hEFhd2 was added with 2 μM hTau40 in the polymerization buffer for direct comparison. At the end of polymerization time, samples were subjected to immunogold labeling, or directly processed for imaging using transmission electron microscopy. All the experiments were repeated at least three independent times.

### Immunogold labeling

To investigate the colocalization of hTau40 and hEFhd2 in aggregate structures, immunogold labeling and electron microscopy was used. Briefly, a parafilm platform was prepared in a humidifying chamber for all incubation steps. Twenty microliters of each sample were fixed with 2% glutaraldehyde (EMS, cat#16120) for 10 min. Then, a 300-mesh carbon-coated nickel grid (EMS, cat#FCF300NI) was placed on a 5 μL drop of each fixed sample spotted on the parafilm for 1 min. Then each grid was rinsed in one 10 μL drop of sterile water that was then wicked away using Whitman filter paper (Capillary Blotting and Wicking applications, cat#GB003). This step was repeated with a 20 μL drop of blocking solution (5% normal goat serum +0.1% bovine serum albumin in TBS). Next, the grids were placed over a 20 μL drop blocking solution for 30 min blocking at room temperature. After blocking, each grid was incubated with a 20 μL drop of primary antibodies for 1.5 h at room temperature. Primary antibody solution was a mixture of Tau 13 1:2500 (Biolegend, cat# 835201 and EFhd2 1:10, rabbit (Prosci, cat# 5657) diluted in TBS/ 5% normal goat serum. After incubation, grids were rinsed with sterile filtered TBS three times 1 min each. The grids were then incubated with a 20 μL drop of secondary antibodies mixture for 1 h at room temperature. Secondary antibodies were 15 mm gold-conjugated goat anti-rabbit (EMS, cat# 25112) and 6 nm gold-conjugated goat anti-mouse (EMS, cat# 25124) diluted in TBS/ 5% NGS 1:20. Subsequently, grids were washed with TBS six times 1 min each. Lastly, the grids were rinsed with a 10 μL drop of water followed by another rinse with a 10 μL drop of VitroEase (2% methylamine vanadate, Thermo Scientific, cat# A51037)). The last step was staining the grid on a 10  μL drop of VitroEase for 2 min. Grids were stored in grid boxes to fully dry before taking the micrographs.

### Transmission electron microscopy

TEM was used to visualize the morphological changes of tau aggregates induced by hEFhd2 (Cox et al., 2016). At the end of the polymerization reaction, unless the samples were processed for immunogold, all samples were processed for TEM using the same procedure. Beforehand, a parafilm platform was prepared on which grid handling took place. We used 300 mesh carbon-coated copper grids (EMS, cat# FCF300-CU). First, 20 μL of each sample were fixed with 2% glutaraldehyde for 10 min. Then, a 5 μL drop of each sample was spotted on the parafilm. The grids were placed on the top of sample drops for 1 min. The grids were rinsed by picking up a 10 μL drop of sterile water and wicking it away using Whatman filter paper. The final step was incubating the grids over a 10 μL drop of 2% uranyl acetate (EMS, cat#22400) for 1 min. The grids were allowed to fully dry in a closed grid box before taking the micrographs using a JEOL JEM-1400 Plus electron microscope at 80 kV and 5,000X and 15,000X magnification (25,000X and 40,000X for immunogold staining). Images were captured with an AMT XR81 digital camera and AMT software version 602.6 (Advanced Microscopy Techniques).

#### Quantitative TEM analysis

Individual aggregate area for all experiments was quantified using Image J (Fiji 2.3) using the images captured at 5000X magnification. First, the scale on Image J was set at 374 pixels equal to 800 nm to match the scale bar. Auto-threshold was selected to differentiate between aggregates versus background. To ensure unbiased detection of aggregated structures, the images of hTau40^m^ (no aggregates) were used to establish the minimum area of true aggregates and to eliminate background of detected specks. Data were compiled from at least three replicates of each experiment using three randomly selected fields of each replicate.

Oligomeric EFhd2 short filaments were quantified, and their average area was set as a baseline (1,500 nm^2^) above which hTau40^m^/hEFhd2 aggregate area was analyzed and counted. The reason is that in hTau40^m^/hEFhd2 samples we observed amorphous aggregates and short filaments that could be ascribed to hEFhd2 oligomerization. Likewise, average area of hTau40^ARA^ filaments were set as a baseline (2000 nm^2^) above which hTau40^ARA^/hEFhd2 aggregate area were analyzed and counted. It is noteworthy that the data, often, had to be curated manually if the software recognized one object as two separate objects, or if it counted a hole as an object. Micrographs with the lowest number of outliers were selected to make the figures presented in the paper.

### Sandwich ELISA

As described in Combs et al. (2017), a nondenaturing sandwich ELISA assay (sELISA) is instrumental to quantify tau oligomeric modifications in disease brains and recombinant protein (Combs et al., 2017). Herein, a slightly modified version of the assay was used to assess EFhd2-induced aggregates formed with hTau40^m^ and hTau40^ARA^. Unless stated otherwise, all steps were undertaken at room temperature with shaking at 200 rpm. Washing and blocking were performed using 200 μL/well. All other steps performed using 50 μL/well. The capture antibodies used were Tau13, TOC1, or Alz50. Tau13 (Biolegend, cat# 835201) is a pan-tau monoclonal mouse IgG1antibody that reacts with monomeric and aggregated tau. TOC1 is a monoclonal mouse IgM antibody that was developed against tau dimers. It is a conformation-dependent antibody whose epitope is presumably revealed with dimerization and oligomerization (RRID#: AB_2832939; Kanaan Lab) (Patterson et al., 2011; Ward et al., 2013). Alz50 is another tau conformation-specific antibody. It is monoclonal mouse IgM that recognizes discontinuous epitope in misfolded tau 2–10 aa and 312–342 aa (RRID#: AB_2313937; Davies lab) (Wolozin et al., 1986; Ksiezak-Reding et al., 1988, 1995; Goedert et al., 1991; Carmel et al., 1996). At the end of overnight incubation of polymerization reaction, samples were initially diluted in 1X PBS to 40 nM (Tau13), 20 nM (TOC1, in presence of ARA), 40 nM (TOC1, in the absence of ARA; and Alz50). High binding 96-well plates (Corning, cat# 3590) were coated with TOC1, Tau13, or Alz50 diluted to 2 ng/μL in 1X PBS and incubated overnight at 4°C. Additional wells were coated with only 1X PBS and were used as a negative antibody control. Sample wells were strictly washed twice with ELISA wash buffer (100 mM borate acid, 25 mM sodium borate, 75 mM NaCl, 0.25 mM thimerosal, 0.4% (w/v) bovine serum albumin, 0.05% (v/v) Tween-20) and then blocked for 1 h with 5% non-fat dry milk (NFDM) prepared in ELISA wash buffer. Then, sample wells were carefully washed twice with ELISA wash buffer followed by adding the diluted samples for 1.5 h. Sample wells were washed 4 times with ELISA wash buffer and incubated with the detection antibodies; rabbit polyclonal pan-tau R1 (RRID#: AB_2832929; Kanaan lab) at 1:10 k for 1.5 h. Afterwards, sample wells were carefully washed 4 times and incubated with goat-anti-rabbit-HRP at 1:5000 (Vector Labs, cat# PI-1000) for 1 h. Wells were washed 4 times before developing with 3,3′,5,5′-tetramethylbenzidine (TMB, Sigma, cat# T8665) 8 min (Tau13, Alz50 and TOC1). Reactions were stopped using 3.5% sulfuric acid. Absorbance readings were measured at 450 nm on a SpectraMax Plus 384 microplate reader (Molecular Devices). Absorbance values of no capture negative control were first subtracted for sample values; then, the background-corrected values were converted to percent light absorbed using the equation $\%A=\left(1-10^{-A}\right)*100$ , where A is equal to absorbance at 450 nm.

### Statistical analysis

All data were analyzed using GraphPad Prism v9.5 (San Diego, www.graphpad.com, RRID:SCR_002798). Before running statistical analysis, outliers were detected and removed from the data. ROUT method with false discovery rate of 1% was used for detection of outliers. Normal distribution was tested using the Shapiro–Wilk test. For comparisons between two groups, unpaired T-test and Mann–Whitney test were used for Gaussian and non-Gaussian distribution samples, respectively. For multiple groups, Kruskal-Wallis test (followed by Dunn’s multiple comparison *post hoc* test) was used for non-Gaussian samples. *p*-Values were calculated with a 95% confidence internal, if nothing is mentioned, it is nonsignificant: otherwise, *p < 0.05, **p < 0.01, ***p < 0.001, ****p < 0.0001. Data presented were shown as mean ± SEM.

## Results

### Incubating EFhd2 with monomeric and filamentous tau resulted in aggregate formation

Previously, our research established the connection between EFhd2 and pathological tau in both a transgenic tau model and postmortem brain tissues of tauopathies (Vega et al., 2008; Ferrer-Acosta et al., 2013b). Furthermore, we demonstrated that EFhd2 influences the conformation of tau by increasing its β-sheet structure (Vega et al., 2018). In addition, EFhd2 has been shown to affect tau’s liquid–liquid phase separation by promoting the formation of solid-like structures *in vitro* (Vega et al., 2019). However, whether EFhd2 can directly drive further aggregation of either monomeric or filamentous tau has yet to be investigated.

In our experimental approach, we utilized arachidonic acid (ARA)-induced tau fibrillization as an *in vitro* model of tau filaments and oligomers (Combs et al., 2017). The polymerization reaction proceeded for 16–18 h at room temperature using either a single recombinant protein or by co-incubating equimolar concentrations of a protein mixture. Subsequently, each reaction was fixed and placed on carbon-coated copper grids for visualization via transmission electron microscopy (TEM). For simplicity, hTau40^m^ refers to full length human tau hTau40 incubated overnight at room temperature without inducer, while hTau40^ARA^ refers to hTau40 incubated overnight with ARA. Additionally, hTau40^m^/hEFhd2 and hTau40^ARA^/hEFhd2 refer to the co-incubation of recombinant human EFhd2 with hTau40 in the absence and presence of ARA, respectively.

**Figure 1:**
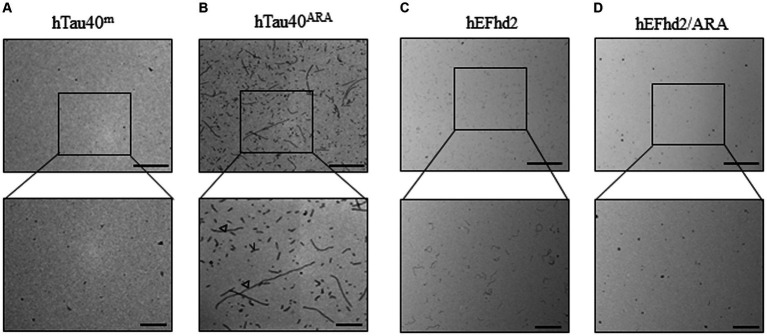
hEFhd2 and hTau40 recombinant proteins incubated in absence or presence of Arachidonic acid (ARA). **(A)** Representative micrograph of monomeric tau incubated overnight in the absence of ARA (hTau40^m^); no obvious aggregates or filaments are formed. **(B)** Representative micrograph of filamentous/oligomeric tau (hTau40^ARA^) by incubating hTau40 with ARA overnight; combination of oligomers (asterisk), short (inverted triangle) and long filaments (open arrowheads) are detected. **(C)** Representative micrograph of overnight polymerization of hEFhd2; short filaments are detected. **(D)** Representative micrograph hEFhd2 polymerization overnight in the presence of ARA (hEFhd2/ARA); remarkable reduction in hEFhd2 filaments is noticed. Scale bar for the top micrographs 800 nm and for the bottom micrographs 200 nm. Experiments were repeated at least three independent times.

To establish a reference to which we could compare the co-incubation of EFhd2 and hTau40, each of the two proteins was first incubated separately both in the absence and presence of ARA. In line with previous research findings, monomeric hTau40 (hTau40^m^) incubated overnight at room temperature without ARA exhibited no detectable filaments or aggregates (Figure 1A) (Kuret et al., 2005; Patterson et al., 2011; Cox et al., 2016). In contrast, Figure 1B shows that overnight incubation of hTau40 in the presence of ARA induced the formation of oligomers (asterisk), as well as short (inverted triangle) and long (open arrowhead) filaments (hTau40^ARA^). Our prior studies established that EFhd2 self-oligomerizes without a nucleation factor or external inducer (Ferrer-Acosta et al., 2013a). Consistently, EFhd2 incubated overnight at room temperature formed short filamentous structures (Figure 1C). However, the addition of ARA reduced EFhd2 filament formation, as seen in Figure 1D. These findings align with our previously reported observations regarding the impact of heparin on EFhd2 self-oligomerization (Ferrer-Acosta et al., 2013b).

**Figure 2:**
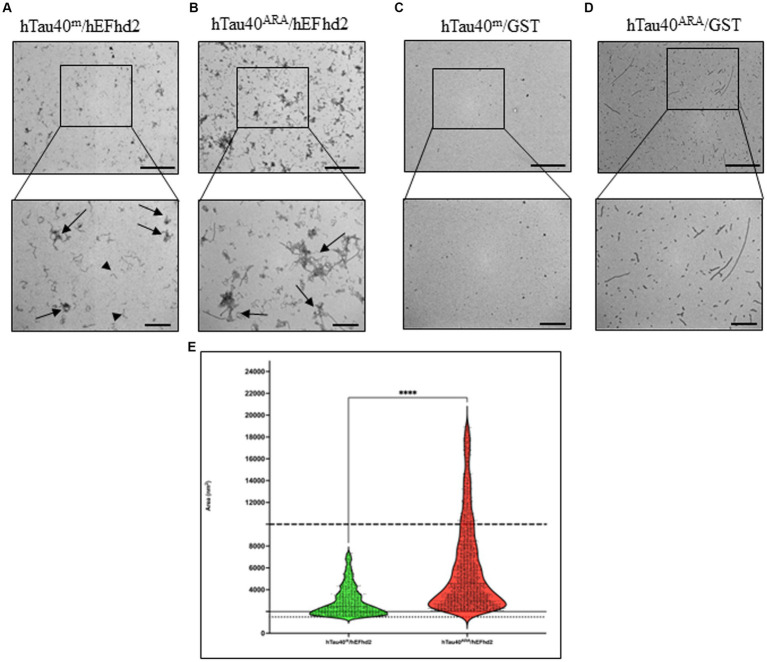
hEFhd2 promotes the aggregation of monomeric and filamentous htau40 *in vitro*. **(A)** Representative micrograph of co-incubation of hEFhd2 (2 μM) hTau40^m^ (monomeric tau) overnight in the absence of ARA; amorphously shaped aggregates are detected (arrows) while EFhd2 oligomeric filaments can be seen (arrowheads). **(B)** Representative micrograph of overnight co-incubation of hEFhd2 with hTau40 in presence of ARA (hTau40^ARA^/hEFhd2); filaments are entangled into larger aggregates (arrows). **(C)** Representative micrograph of co-incubation of GST with hTau40^m^ overnight in the absence of ARA (hTau40^m^/GST); no aggregates or filaments were detected. **(D)** Representative micrograph of overnight co-incubation of GST with hTau40 in the presence of ARA (hTau40^ARA^/GST), similar to hTau40^ARA^, a combination of oligomeric and filamentous tau exists. Scale bar for the top micrographs 800 nm and for the bottom micrographs 200 nm. Experiments were repeated at least three independent times. **(E)** Quantitative EM analysis of individual aggregate area shows that hTau40^ARA^/hEFhd2 aggregates are significantly larger than hTau40^m^/hEFhd2 aggregates. Data are represented in violin blot to show the distribution of individual aggregate area. Data were drawn from n = 3 replicates/group and 3 micrographs for each replicate. Number of outliers detected and excluded are 199 and 380 for hTau40^m^/hEFhd2 and hTau40^ARA^/hEFhd2, respectively. Analysis was conducted by Mann–Whitney test, ****p < 0.0001. Values are presented as mean ± SEM. The dotted and solid lines represent the average aggregate area of hEFhd2 filaments and hTau40^ARA^ filaments used as baseline to quantify hTau40^m^/hEFhd2 and hTau40^ARA^/ hEFhd2 aggregates, respectively. The dashed line represents the average individual aggregate area of tau long filaments.

EFhd2 co-incubation with hTau40^m^ and hTau40^ARA^ led to the formation of distinct protein aggregates (Figure 2). EFhd2 induced the formation of amorphous aggregates when added to hTau40^m^. Those aggregates were not observed in any of the recombinant proteins alone (Figure 2A, arrows; compared with Figures 1A,C). In addition, short filaments were noticed surrounding the larger protein aggregates (Figure 2A, arrowheads). These short filaments could represent EFhd2 self-oligomerization, as observed in Figure 1C. In contrast, when hEFhd2 was added to hTau40 in the presence of ARA (hTau40^ARA^/hEFhd2), we detected aggregates that are different from those observed in hTau40^m^/hEFhd2 (Figure 2A vs. Figure 2B). The observed aggregates in hTau40^ARA^/hEFhd2 seem to be formed through coalescing filaments together in a way that forms proteins tangled or intertwined together (arrows in Figure 2B). The protein aggregates observed in hTau40^ARA^/hEFhd2 are clearly different from hTau40^ARA^ filaments (Figure 1B).

Quantitatively, hTau40^ARA^/hEFhd2 and hTau40^m^/hEFhd2 aggregates are significantly different in area (Figure 2E). As described in the methods section, we quantified the average area for EFhd2 filaments (Figures 1C, 2E, dotted line) and hTau40^ARA^ filaments (Figures 1B, 2E, solid line) and used them as a baseline above which we calculated the area of the protein aggregates observed in hTau40^m^/hEFhd2 and hTau40^ARA^/hEFhd2, respectively. We also subtracted the detected electron dense speckles that represent artifacts of the staining process. The distribution of hTau40^ARA^/hEFhd2 aggregate areas highlights the formation of larger aggregates than the average area of long tau filaments in hTau40^ARA^ (Figure 2E, dashed line), indicating that the overnight co-incubation of hEFhd2 and hTau40 in the presence of ARA led to the formation of larger distinct protein aggregates. In addition, the area of hTau40^m^/hEFhd2 aggregates is larger than the area of the EFhd2 filaments (Figure 2E, dotted line). These results indicate that incubation of EFhd2 with either hTau40^m^ or hTau40^ARA^ induces the formation of protein aggregates with different structural characteristics.

EFhd2 protein bears a net negative charge. Hence, it was plausible to ascribe the observed protein structures formed by incubating hEFhd2 with hTau40 in the absence or presence of ARA to mere electrostatic interaction between the two proteins. Alternatively, EFhd2 in equimolar concentration might create a crowded environment that could exert changes on tau folding and promote the formation of the observed aggregates. To address those two explanations, we conducted a control experiment using GST protein. We chose GST because it shares some physicochemical properties with EFhd2 (i.e., molecular weight and isoelectric point). Importantly, GST has not been shown to be associated with tau. Thus, we co-incubated recombinant GST with hTau40 in the absence or presence of ARA (Figures 2C,D). GST neither promoted changes in hTau40^m^ nor did it induce similar structures like those formed with hEFhd2 (Figure 2A vs. Figure 2C). Likewise, adding GST to hTau40 in the presence of ARA did not lead to the formation of large protein aggregates as observed when hEFhd2 (Figure 2B vs. Figure 2D). These results strongly support the argument that the protein aggregates observed during the co-incubation of hEFhd2 with hTau40, with or without ARA, can be clearly attributed to the protein–protein interaction between EFhd2 and tau.

### hEFhd2 and hTau40 colocalize in the newly formed protein aggregates

**Figure 3:**
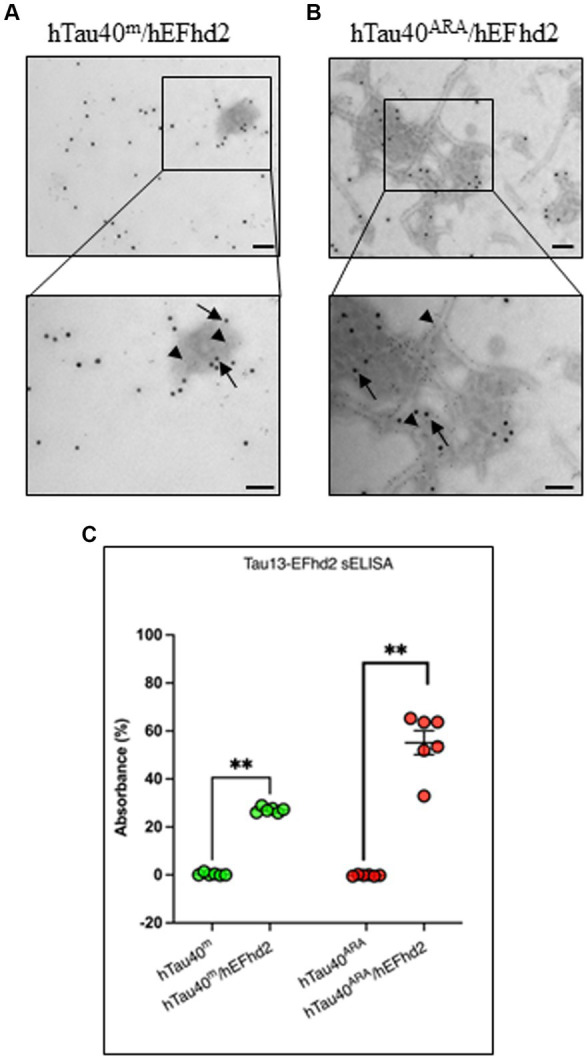
hEFhd2 and hTau40 colocalize on hEFhd2-induced aggregates. After overnight polymerization of recombinant proteins, all samples were labeled using both Tau 13 antibody (IgG1 mouse antibody) and anti-EFhd2 (rabbit antibody). Distinct co-labeling was confirmed using gold-conjugated secondary antibodies anti-mouse 6 nm (small gold particles) and anti-rabbit 15 nm (large particles). **(A)** Representative micrograph of immunogold labeling conducted on hTau40^m^/hEFhd2 (without ARA). Co-labeling of EFhd2 (large particles; arrows) and tau (small particles; arrowheads) was detected on the aggregates. **(B)** Representative micrograph of immunogold labeling conducted on hTau40^ARA^/hEFhd2 (with ARA). Co-labeling of EFhd2 (large particles; arrows) and tau (small particles; arrowheads) was detected on the observed aggregates of entangled filaments. Scale bar for the top and for bottom micrographs are 100 nm. Experiments were repeated at least three independent times. **(C)** sELISA was conducted using Tau13 as capture antibody with anti-EFhd2 as detection antibody. hTau40^m^/EFhd2 and hTau40^ARA^/hEFhd2 samples show increased signals compared to their respective controls using Mann–Whitney test; p** < 0.01. Values are presented as mean ± SEM.

To determine the colocalization of both hEFhd2 and hTau40 within the observed aggregates, immunogold electron microscopy was employed. The detection of hTau40 and hEFhd2 was carried out using Tau13 and anti-EFhd2 antibodies, respectively, immediately after the overnight polymerization reaction. Control experiments were initially conducted to demonstrate the specificity of primary antibodies (Supplementary Figures S1, A–C) and secondary antibodies (Supplementary Figure S1D) used in the immunogold labeling. Figure 3A illustrates that both hTau40 (small particles, arrowheads) and hEFhd2 (large particles, arrows) colocalize on the same amorphous protein aggregated structure. The electron density of amorphous protein aggregates can be challenging to focus when using immunogold labeling in electron microscopy. The lack of defined structural features and the uneven distribution of electron-dense material within amorphous aggregates affects the clarity of the imaging, including the precise localization of immunogold labels. Therefore, focusing on amorphous aggregates is more challenging compared to well-defined structures with clear boundaries. Consistently, colocalization of hTau40 (small particles, arrowheads) and hEFhd2 (large particles, arrows) was evident in hTau40^ARA^/hEFhd2 (Figure 3B). Interestingly, hEFhd2 was detected in the protein dense area where hTau40 filaments are coalescing into the aggregates (Figure 3B). These results endorse that the observed aggregates comprise hEFhd2 and hTau40.

The aforementioned results along with previous studies confirm the direct association between hEFhd2 and hTau40. Nonetheless, to rule out that antibody binding to the protein aggregates are technical artifacts (e.g., due to sample fixation with glutaraldehyde), we opted to use nondenaturing sELISA as an additional approach to verify the association of hEFhd2 and hTau40 on the same structures. The assays were conducted using Tau13 as capture antibody and anti-EFhd2 as detection antibody. Control samples were hTau40 in the absence (hTau40^m^) or presence (hTau40^ARA^) of ARA. As demonstrated in Figure 3C, EFhd2 signal was not detected in these samples. In contrast, EFhd2 signal was detected in both hTau40^m^/hEFhd2 and hTau40^ARA^/hEFhd2 samples. The data collectively attest to the protein–protein interaction between hTau40 and hEFhd2.

### The formation of hEFhd2-hTau40 aggregates is contingent on the concentration of EFhd2

**Figure 4:**
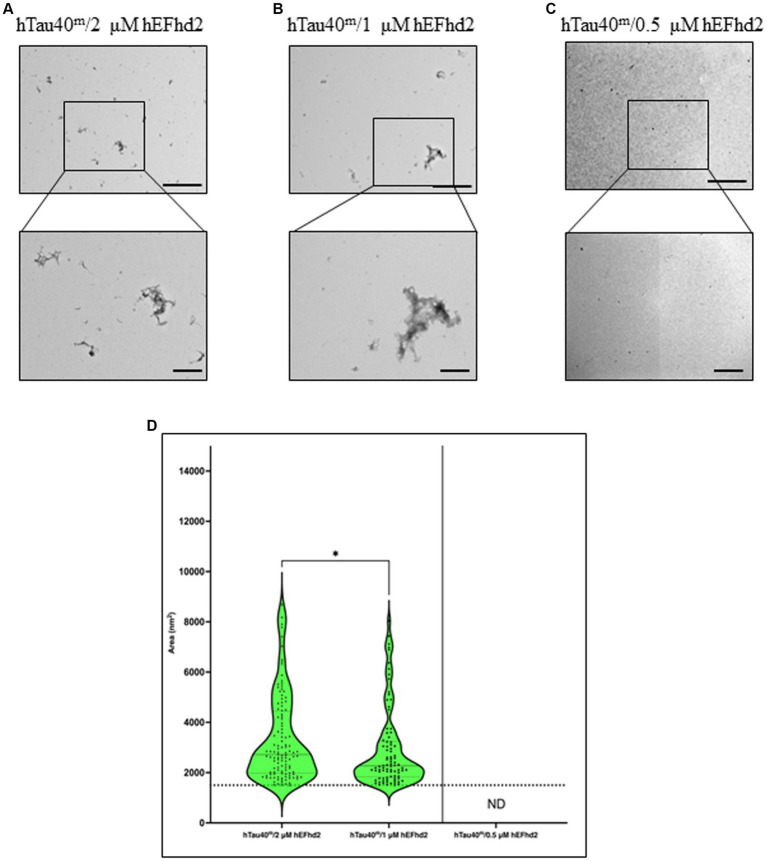
The formation of hTau40^m^/hEFhd2 amorphous aggregates is hEFhd2 concentration dependent. **(A–C)** Representative micrographs of hTau40^m^/hEFhd2 (co-incubating 2 μM of hTau40 and 2 μM hEFhd2) **(A)**, 1 μM hEFhd2 **(B)**, or 0.5 μM hEFhd2 **(C)** overnight in the absence of ARA. **(D)** Quantitative EM analysis of individual aggregate area of hTau40^m^/hEFhd2 aggregates represented as violin blot. Because 0.5 μM hEFhd2 failed to promote perceptible aggregation (above the baseline of EFhd2 oligomeric filaments area; dotted line), it was not included in the analysis. The outliers detected and excluded are 23 (hTau40^m^/2 μM hEFhd2) and 20 (hTau40^m^/1 μM hEFhd2). The comparison between 2 and 1 μM hEFhd2-induced aggregates was conducted by Mann–Whitney test, p* < 0.05. Values are presented as mean ± SEM. Dotted line represent the average individual aggregate area of hEFhd2 filaments. Scale bar for the top micrographs 800 nm and for the bottom micrographs 200 nm.

To further investigate the extent of hEFhd2-hTau40 aggregation, we explored whether the formation of these aggregates relies on the concentration of hEFhd2. When equimolar hEFhd2 and hTau40^m^ were used, the expected aggregates formed (compare Figures 2A, 4A). Reducing hEFhd2 concentration by half to 1 μM showed minimal differences in the aggregate structure of hTau40^m^/hEFhd2 (Figure 4B). However, quantitative analysis revealed that 1 μM hEFhd2 led to the formation of aggregates with significantly smaller areas compared to 2 μM hEFhd2 (Figure 4D). Importantly, it should be noted that, as described earlier (Figure 2E), hTau40^m^/hEFhd2 aggregates had a larger area than the area of hEFhd2 self-oligomeric filaments baseline (Figure 4D, dotted line). Conversely, with 0.5 μM hEFhd2, no aggregates were detected above the baseline (Figure 4C). These results indicate that the formation of hTau40^m^/hEFhd2 aggregates is indeed hEFhd2 concentration dependent.

**Figure 5:**
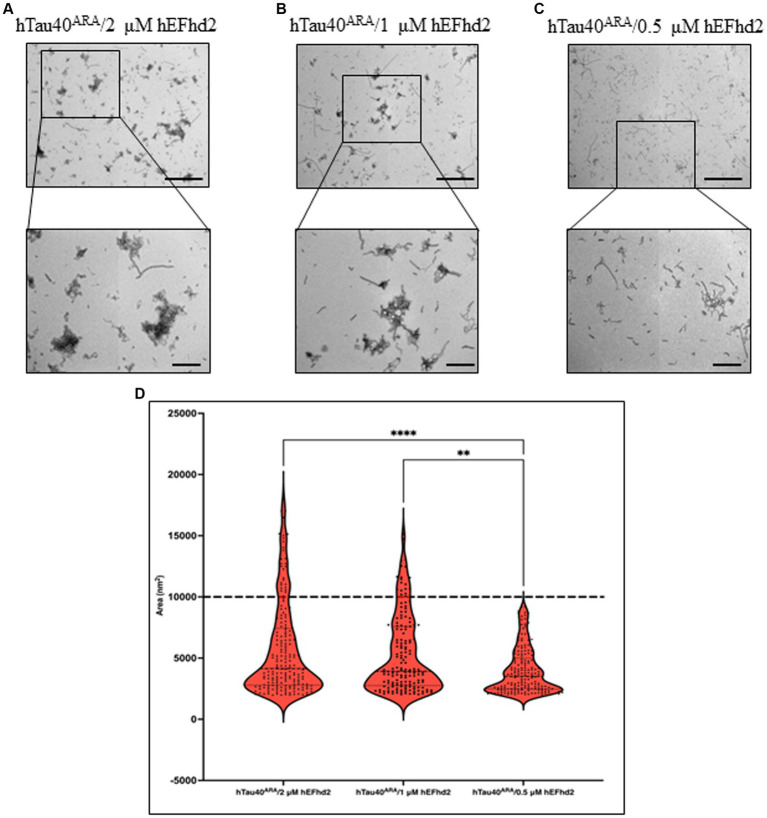
hTau40^ARA^/hEFhd2 entangled structure formed in hEFhd2 concentration-dependent manner. **(A-C)** Representative micrographs of hTau40^ARA^/hEFhd2 by co-incubation of 2 μM of hTau40 and 2 μM hEFhd2 **(A)**, 1 μM hEFhd2 **(B)**, or 0.5 μM hEFhd2 **(C)** overnight in the presence of ARA. **(D)** Quantitative EM analysis of individual aggregate area of hTau40^ARA^/hEFhd2 aggregates. The outliers detected and excluded are 40 (hTau40^ARA^/2 μM hEFhd2), 26 (hTau40^ARA^/1 μM hEFhd2), and 27 (hTau40^ARA^/0.5 μM hEFhd2). Kruskal-Wallis was conducted for statistical comparison between groups. Dunn’s test was used for *post hoc* multiple comparison **p < 0.01, ***p < 0.001. Values are presented as mean ± SEM. Dashed line represent the average individual aggregate area of tau long filaments. Scale bar for the top micrographs 800 nm and for the bottom micrographs 200 nm.

We also examined the effect that different hEFhd2 concentrations have on the formation of aggregates when incubated with hTau40^ARA^ (hTau40 in the presence of ARA). EM micrographs in Figures 5A,B illustrate unnoticeable structural differences in hTau40^ARA^/hEFhd2 when 2 and 1 μM hEFhd2 concentrations were used. In contrast, detection of the hTau40^ARA^/hEFhd2 entangled protein aggregates was remarkably diminished when 0.5 μM hEFhd2 was used and short filaments predominated in the fields (Figure 5C). Moreover, statistical analysis presented in Figure 5D showed a significant difference in aggregate area when either 2 μM or 1 μM hEFhd2 were used in comparison to 0.5 μM hEFhd2. No significant difference was detected in the aggregates area between 2 μM and 1 μM hEFhd2. These results imply that the extent of hEFhd2-hTau aggregation is directly correlated with varying EFhd2 concentrations.

### Influence of hEFhd2 at various stages of hTau40 filament formation *in vitro*

**Figure 6:**
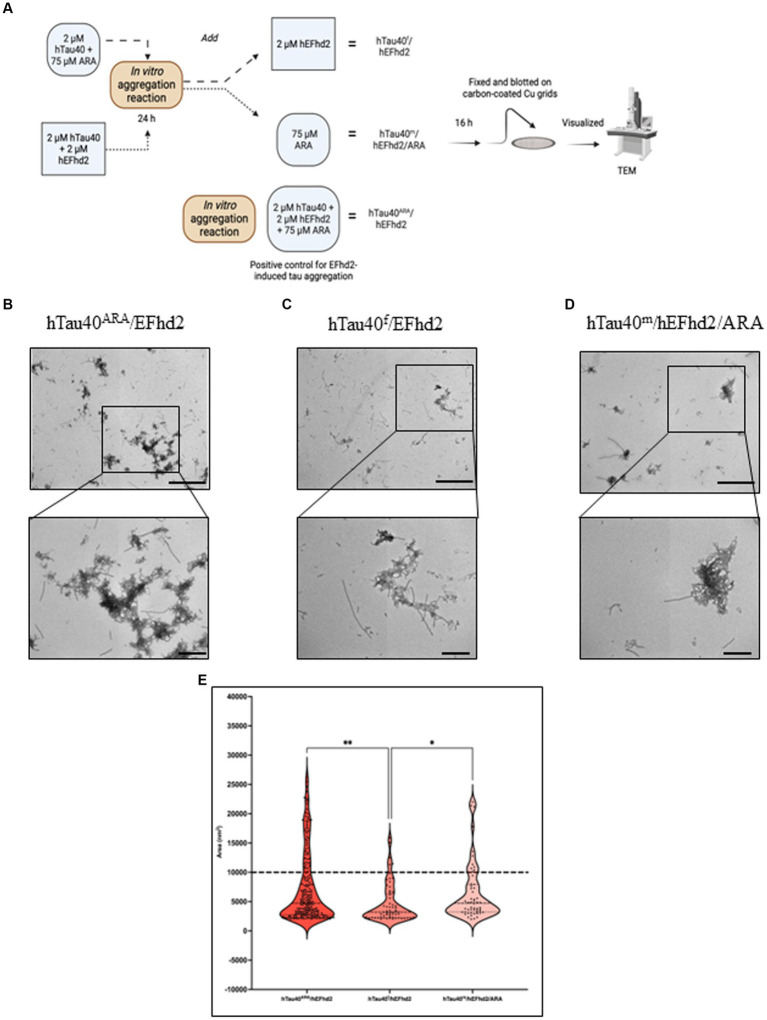
Influence of hEFhd2 at various phases of hTau40 filaments formation *in vitro*. **(A)** Summary of experimental paradigm. The figure summarizes the polymerization reaction conducted for this experiment and the terminology used to indicate each sample. All the reactions were conducted using 2 μM of hEFhd2 and hTau40. Arachidonic acid was used as at 75 μM. hTau40^f^/hEFhd2 sample is incubating hTau40 and ARA for 24 h followed by adding hEFhd2 and let the reaction proceed for another 16 h. hTau40^m^/hEFhd2/ARA sample is co-incubating hEFhd2 and hTau40 for 24 h then ARA was added, and the reaction proceeded for 16 h. hTau40^ARA^/hEFhd2 sample is co-incubating hEFhd2 and hTau40 in the presence of ARA for 16 h. Then all samples were fixed on grids and visualized with TEM. This illustration was created with Biorender.com. **(B)** Representative micrograph of hTau40^ARA^/hEFhd2 showing hTau40 filaments entangled into larger aggregates. **(C)** Representative micrograph of hTau40^f^/hEFhd2 where the micrographs were taken after the 40-h reaction. A clear reduction in size and number of aggregates was noticed. **(D)** Representative micrograph of hTau40^m^/hEFhd2/ARA where micrographs were taken after the 40-h reaction. A tangible reduction in the size and number of aggregates were observed from the micrographs. **(E)** Quantitative EM analysis of individual aggregate area shows that aggregates of hTau40^ARA^/hEFhd2 and hTau40^m^/hEFhd2/ARA aggregates are significantly higher than hTau40^f^/hEFhd2 aggregates. No significant difference was detected between hTau40^ARA^/hEFhd2 and hTau40^m^/hEFhd2/ARA. Outliers were identified first using ROUT method with false discovery rate 1%. The outliers detected and excluded are 44 (hTau40^ARA^/hEFhd2), 12 (hTau40^f^/hEFhd2), and 14 (hTau40^m^/hEFhd2/ARA). Then, Kruskal-Wallis was conducted for statistical comparison between groups. Dunn’s test was used for *post hoc* multiple comparison. * < 0.05, ** < 0.01. Values are presented as mean ± SEM. Dashed line represent the average individual aggregate area of tau long filaments. Scale bar for the top micrographs 800 nm and for the bottom micrographs 200 nm.

The co-incubation of hEFhd2, hTau40, and ARA (a robust inducer of tau fibrillization) elicited the entanglement of hEFhd2 with hTau40 filaments, suggesting that hEFhd2 did not interfere with ARA-induced hTau40 filament formation (Figure 2B). Figure 6A summarizes the experimental paradigm followed to examine how the addition of hEFhd2 to hTau40 prior to or after ARA impacts formation of protein aggregates. In Figure 6C, hTau filaments (hTau40^f^) were generated by initially incubating hTau40 with ARA for 24 h, followed by incubation with hEFhd2 for additional 16 h (hTau40^f^/hEFhd2). Conversely, in Figure 6D, we incubated hEFhd2 and hTau40 (hTau40^m^/hEFhd2) for 24 h, followed by ARA for additional 16 h (hTau40^m^/hEFhd2/ARA). TEM was used to validate the formation of hTau40 filaments after 24 and 40 h incubation. The micrographs show that hTau40^f^ at 24 h (before adding hEFhd2) and at 40 h (the total experimental duration) are virtually the same as hTau40^ARA^ formed overnight (Supplementary Figures S2, A–C). Similarly, we did not notice a difference between hTau40^m^/hEFhd2 at 24 or 40 h and hTau40^m^/hEFhd2 after overnight reaction (Supplementary Figures S2, D,E). Figure 6B shows the consistent formation of hTau40^ARA^/hEFhd2 aggregates as a reference.

Introducing hEFhd2 after ARA-induced tau filament formation (hTau40^f^/hEFhd2) comparatively reduced the formation of intertwined filamentous structures (Figure 6B vs. Figure 6C). Additionally, isolated filaments were visible alongside the intertwined filamentous structures (Figure 6C). The quantitative analysis showed a significant decrease in the area of hTau40^f^/hEFhd2 entangled filamentous structures in comparison to the area of structures that emerged when all three components were co-incubated simultaneously hTau40^ARA^/hEFhd2 (Figure 6E). It is important to note that despite the differences in area, the observed intertwined filamentous structures seem morphologically similar (Figures 6B,C).

These results indicate that hEFhd2 may be more effective at entangling hTau40 filaments during their formation rather than after they are fully formed. In other words, hEFhd2 may not interfere with the initial formation of hTau40 filaments; rather, it promotes their entanglement as they are being generated. Along this line of thinking, pre-incubation of hTau40 and hEFhd2 (hTau40^m^/hEFhd2) did not interfere with ARA-induced hTau40 filament formation (Figure 6D). Notably, filamentous structures radiate from the amorphous protein aggregates when hTau40 and hEFhd2 were incubated before the addition of ARA (Figure 6D). The observed structures are generally similar to those shown in Figures 6B,C. Moreover, the area of aggregates formed when ARA was added after the incubation of hEFhd2 and hTau40 (hTau40^m^/hEFhd2/ARA) showed no significant difference from those observed when all three components were co-incubated (hTau40^ARA^/hEFhd2) (Figure 6E). Taken together, these results signal that hEFhd2 does not interfere with the ARA-induced formation of hTau40 filaments. We can infer that hEFhd2 could be more effective at entangling tau filaments during ARA-induced tau polymerization than after long hTau40 filaments have been fully formed.

### Assessment of hEFhd2-hTau aggregates with tau-conformation specific antibodies

Tau conformational changes are among the pivotal pathological events associated with neuronal toxicity (Brunden et al., 2008; Spires-Jones et al., 2009; Lasagna-Reeves et al., 2012; Cowan and Mudher, 2013; Chung et al., 2021). Hence, several tools have been geared toward characterizing tau conformation associated with its aggregated forms to understand their spatial and temporal evolution during tauopathies. Tau oligomeric complex 1 (TOC1) antibody is a conformation-dependent antibody that recognizes tau oligomers (Patterson et al., 2011; Ward et al., 2013). TOC1 epitope is exposed upon oligomerization and presumably is masked with further tau fibrillization. Sandwich ELISA (sELISA) is used to quantify and characterize TOC1 signal in tauopathies and *in vitro* hTau fibrils (Tiernan et al., 2016; Combs et al., 2017). Thus, sELISA was conducted here to determine whether hEFhd2-hTau aggregates have conformational changes detected by TOC1.

**Figure 7:**
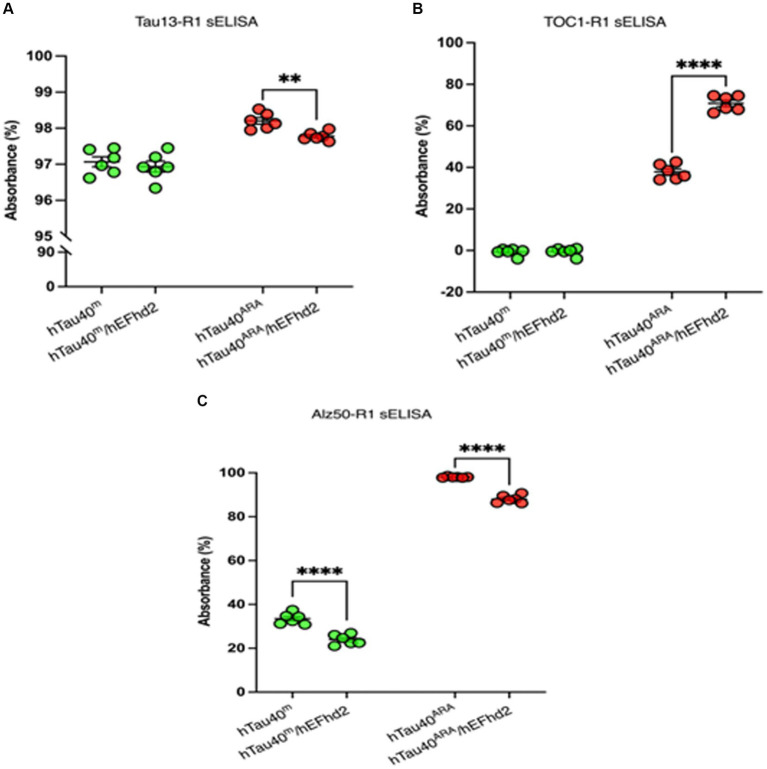
Assessment of hTau40/hEFhd2 aggregates with hTau-conformation specific antibodies. Sandwich ELISA was done after overnight polymerization reaction using 2 μM recombinant proteins. It is important to note that samples prepared in the absence of ARA (hTau40^m^/hEFhd2 and hTau40^m^) were assessed by sELISA separately from those in the presence of ARA (hTau40^ARA^/hEFhd2 and hTau40^ARA^). Therefore, no direct statistical comparison between the two sets was conducted. **(A)** Total hTau40 was assessed using Tau13 as capture antibody and R1 as detection antibody. Unpaired T-test revealed no significant difference in tau level between hTau40^m^/hEFhd2 and hTau40^m^. However, hTau40 level in hTau40^ARA^/hEFhd2 is significantly lower than hTau40^ARA^ analyzed by unpaired T-test. **(B)** Oligomeric hTau40 conformation was assessed using TOC1as capture antibody and R1 as detection antibody. Unpaired T-test showed that hTau40^ARA^/hEFhd2 has higher signal compared to hTau40^ARA^. **(C)** sELISA using Alz50 as capture antibody and R1 as detection antibody. Unpaired T-test revealed a significant difference between hTau40^m^/hEFhd2 and hTau40^m^. Likewise, Alz50 reactivity was significantly lower in hTau40^ARA^/hEFhd2 compared to hTau40^ARA.^ *p < 0.05, ****p < 0.0001. Values are presented as mean ± SEM. The data were drawn from *n* = 6.

hTau40^m^/hEFhd2 and hTau40^ARA^/hEFhd2 aggregates were formed as described earlier and validated using TEM. The protein aggregates were then subjected to sELISA. As illustrated in Figure 7A, sELISA using Tau13 as capture antibody and R1 (pan-tau) as detection antibody was conducted to detect total hTau40 (monomeric and aggregated) levels. The results point to nearly comparable tau levels in hTau40^m^/hEFhd2 and hTau40^m^. In contrast, a significantly lower hTau40 level is detected in hTau40^ARA^/hEFhd2 compared to hTau40^ARA^.

We assessed TOC1 reactivity of aggregated hTau40 in the presence of hEFhd2. As expected, TOC1 showed no affinity for hTau40^m^ (monomeric tau without ARA), which served as a negative control (Figure 7B). Consistent with previous research (Tiernan et al., 2016; Combs et al., 2017), TOC1 successfully captured hTau40^ARA^, confirming the formation of ARA-induced hTau40 oligomers and the associated conformational changes (Figure 7B). Interestingly, hTau40^m^/hEFhd2 (hEFhd2 and hTau40 in the absence of ARA) was not captured with TOC1 (Figure 7B). This result indicates that the formation of hTau40^m^/hEFhd2 aggregates does not involve the conformation change detected by TOC1. Alternatively, amorphous protein aggregate might mask the conformational epitope. Conversely, TOC1 reactivity in hTau40^ARA^/hEFhd2 (hEFhd2 and hTau40 in the presence of ARA) was significantly higher compared to hTau40^ARA^ (Figure 7B). These results indicate that TOC1 captures hTau40^ARA^/hEFhd2 aggregates more effectively than hTau40^ARA^. That could imply that ARA-induced hTau filament formation in the presence of hEFhd2 leads to greater exposure of the TOC1 specific conformational epitope in comparison to hTau40^ARA^. It is important to note here that statistical analysis was not conducted to compare between samples in the absence of ARA to samples in the presence of ARA (See Material and Methods).

We also tested if hEFhd2 induces a tau conformational change that could be detected by Alz50. Alz50 antibody recognizes a discontinuous epitope that involves tau’s N-terminus and microtubule-binding repeat domains, which is formed due to a conformational change associated with tau oligomer formation (Wolozin et al., 1986; Ksiezak-Reding et al., 1988; Goedert et al., 1991; Ksiezak-Reding et al., 1995; Carmel et al., 1996). Following the same methodology, we conducted sELISA to analyze Alz50 reactivity. Figure 7C demonstrates that Alz50 captured hTau40^ARA^. Alz50 signal was also detected with hTau40^m^, suggesting that during the incubation time some monomeric hTau40 adapted the conformational change detected by Alz50. The incubation of hEFhd2 with hTau40 in the absence (hTau40^m^/hEFhd2) or presence (hTau40^ARA^/hEFhd2) of ARA significantly reduced Alz50 reactivity compared to hTau40^m^ and hTau40^ARA^, respectively. Reduced Alz50 signal in hTau40^ARA^/hEFhd2 could be due to the formation of a different tau conformation that masks the Alz50-epitope.

## Discussion

In this study, we demonstrate that EFhd2 has the capacity to promote tau aggregation forming a unique higher order structure. Currently, the literature abounds with several studies investigating the effect of other proteins on pathological tau formation. Nonetheless, EFhd2 is the first to show the propensity to entangle tau filaments into larger aggregates.

Understanding tau pathogenesis is a crucial step toward the identification of effective treatments of tauopathies, such as AD. However, the mechanisms that lead to the formation of pathological tau species *in vivo* remain to be elucidated. *In vitro* studies provided important information about the biochemical properties of tau proteins and their propensity to form aggregates. Tau proteins do not spontaneously oligomerize *in vitro*. Tau requires external inducers that largely serve as nucleation factors to promote the formation of tau oligomers or filaments (Goedert et al., 1996; Wilson and Binder, 1997; Chirita et al., 2003; Lasagna-Reeves et al., 2010). Therefore, we have been convinced that tau association with other proteins could be playing a major role in tau pathogenesis. Against this backdrop, we discovered the calcium-binding protein EFhd2 as a tau-associated protein in a tauopathy mouse model and AD brain (Vega et al., 2008; Ferrer-Acosta et al., 2013b; Vega, 2016).

In our previous studies, EFhd2 co-immunoprecipitated with pathological tau in brain extracts from AD and other tauopathies (Vega et al., 2008). EFhd2 also colocalized with pathological tau in the somatodendritic compartment (Ferrer-Acosta et al., 2013b). Additionally, immunogold EM analysis of the sarkosyl insoluble fraction of AD frontal cortex confirmed co-labeling of filamentous structures by tau and EFhd2 (Ferrer-Acosta et al., 2013b). That provided further evidence that EFhd2 is associated with tau filamentous structures. However, whether EFhd2 directly binds to tau filaments or influences their formation remains unclear. Furthermore, EFhd2 impacted tau protein dynamics demonstrated by enhancing ThS signal and promoting the formation of solid-like structures in controlled *in vitro* conditions (Vega et al., 2018, 2019).

Thence, we hypothesize that EFhd2 plays a direct role in promoting tau aggregation. To test this hypothesis, we examined EFhd2’s capacity to co-aggregate with monomeric (hTau40^m^) and ARA-induced filamentous (hTau40^ARA^) hTau40 *in vitro*. The results indicate that the presence of hEFhd2 leads to the aggregation of hTau40 even in the absence of ARA. Immunogold analysis revealed that the resulting amorphous protein aggregates consist of both hTau40^m^ and hEFhd2 intricately connected. Furthermore, adding hEFhd2 did not interfere with the ARA-induced formation of hTau40^ARA^ (filaments and oligomers). Significantly, it intertwined with hTau40 filaments into uniquely formed aggregates. Immunogold labeling also demonstrated that hEFhd2 and hTau40 colocalize within these aggregates wherein hEFhd2 predominantly situated at the core connecting the hTau40 filaments.

To affirm the specificity of hEFhd2, we investigated whether GST, a molecule sharing certain physicochemical characteristics with hEFhd2, triggers hTau40 aggregation *in vitro* (Figure 2). The findings revealed that GST did not prompt the formation of aggregates with monomeric or filamentous hTau40. Therefore, we conclude that the impact of EFhd2 on tau aggregation *in vitro* is EFhd2 specific.

EFhd2 self-oligomerizes and forms short filaments without an aggregation inducer. That sparks the possibility that the observed protein aggregates with monomeric tau comprise solely of EFhd2 oligomeric filaments. First, morphologically, EFhd2 filaments are not comparable to hTau40^m^/EFhd2 aggregates (Figure 1C vs. Figure 2A). Second, quantitative analysis revealed that EFhd2-induced aggregates with hTau40^m^ are larger than the average area of EFhd2 filaments (the dotted line in Figure 2E). Therefore, it is reasonable to deduce that those protein aggregates comprise EFhd2 and tau together, as verified with immunogold labeling and sELISA data (Figure 3).

The presented findings raise the question whether EFhd2 filaments are necessary for the formation of aggregates with monomeric and filamentous tau. It should not escape our attention that adding ARA induced a clear reduction on EFhd2 self-oligomerization (Figure 1D). In fact, this is in line with our published research on the effect of heparin on EFhd2 (Ferrer-Acosta et al., 2013b). Heparin and ARA broadly induce *in vitro* aggregation via electrostatic interaction with positively charged proteins (e.g., tau). Given the fact that EFhd2 is a negatively charged molecule, we could speculate that a degree of repulsive force exists between EFhd2 and those aggregation inducers that hinder EFhd2 from self-oligomerization. Overall, the evident reduction in EFhd2 self-oligomerization in the presence of ARA undermines the possibility that the unique entangled hTau40^ARA^/hEFhd2 aggregates necessitate EFhd2 filaments.

Previously, Kayed lab established an alternative method for *in vitro* tau fibrillization using amyloid-β (Αβ) peptide oligomers (Lasagna-Reeves et al., 2010). Like EFhd2, Αβ peptide has a spontaneous propensity to aggregate and form fibrils/oligomers *in vitro*. This characteristic was leveraged to generate *in vitro* tau filaments/oligomers instead of using the conventional polyanionic compounds. In this method, the preformed Αβ oligomers, added at substoichiometric concentration, act as a nucleation seed that promotes tau fibrillization. An *ad hoc* deduction would be that EFhd2 has the same effect on tau fibrillization as Αβ peptides, and that EFhd2 filaments could be seeding tau aggregation *in vitro*. Although we do not rule out this possibility, there is a clear morphological distinction of tau aggregates formed with EFhd2 versus Αβ peptide. Although colocalization was not shown, Αβ oligomers act as a nidus to monomeric tau that becomes misfolded and further aggregates to filaments and oligomers. On the other hand, EFhd2 incubation with tau induced the formation of larger unique, amorphous aggregates wherein EFhd2 and tau colocalize.

Earlier, we demonstrated that EFhd2 impacts β-sheet structure formation of tau *in vitro* (Vega et al., 2018). Therefore, we investigated whether hEFhd2 co-aggregation with hTau induces conformational changes detectable by either TOC1 or Alz50 (Figure 7). TOC1 targets a linear epitope (209–240 aa) exposed during oligomerization (Patterson et al., 2011; Ward et al., 2013). In contrast, Alz50 recognizes a discontinuous epitope involving distant amino acids that come into proximity as a result of conformational changes associated with tau oligomerization (Ksiezak-Reding et al., 1988, 1995; Goedert et al., 1991). The results indicated that hTau40^m^/hEFhd2 did not expose the TOC1-recognized epitope. Conversely, the signal from hTau40^ARA^/hEFhd2 samples was significantly higher than that detected with ARA-induced hTau40^ARA^. On the other hand, Alz50 reactivity diminished in both hTau40^ARA^/hEFhd2 and hTau40^m^/hEFhd2. Another important observation is the reduced Tau13 reactivity to hTau40^ARA^/hEFhd2 compared to hTau40^ARA^ shown in Figure 7A. Taken together, differential reactivity of three tau antibodies that recognize various epitopes speaks to the conformational changes that could be induced by the entanglement of ARA-induced tau filaments in the presence of EFhd2. That leads to enhancing TOC1’s epitope exposure while possibly masking Alz50 and Tau13 epitopes.

Previous studies have shown tau-associated proteins that modulate the formation of tau filaments *in vitro*, such as TIA1, Hsp22, FKBP51, S100B and others (Mandelkow and Mandelkow, 2012; Fontaine et al., 2015; Oroz et al., 2018; Jiang et al., 2019; Darling et al., 2021; Moreira et al., 2021; Moreira and Gomes, 2023). The effect of these tau-associated proteins on tau filament formation has been studied in the presence of either heparin or ARA. Generally, these studies examined whether the tau-associated protein had an impact on altering the size of tau filaments induced by heparin or ARA without necessarily assessing any changes in the structure of tau filaments. Our study contrasts with these previous studies in that we showed that EFhd2 induced monomeric hTau40 aggregation and entangled hTau40 filaments into larger clusters. We showed that EFhd2 and hTau40 colocalize in the detected structures. Additionally, we demonstrated that EFhd2 does not affect ARA-induced tau filament formation. Thus, EFhd2 is a tau-associated protein that induces the formation of entangled tau filaments (Alhadidy and Kanaan, 2024). Nonetheless, it is worth mentioning that pathological tau also undergoes diverse molecular changes, including phosphorylation, acetylation, truncation, and other modifications, which could contribute to its aggregation. Furthermore, EFhd2 is a phosphoprotein and a target of CDK5 (Vazquez-Rosa et al., 2014). Therefore, further inquiry into the impact of phosphorylated EFhd2 on both modified and unmodified tau forms is necessary to further study the effect of EFhd2 on tau protein dynamics. Above all, this study offers an *in vitro* model that could be leveraged to examine the interplay between EFhd2 and different tau isoforms. Nonetheless, assessing the influence of EFhd2 on tau-induced neurotoxicity *in vivo* is a pivotal future direction. These ensuing *in vivo* studies will enhance our understanding of EFhd2’s role in tauopathies and its potential as a target for modulating tau-mediated neurodegeneration.

## Data availability statement

The raw data supporting the conclusions of this article will be made available by the authors, without undue reservation.

## Author contributions

AS: Data curation, Formal analysis, Investigation, Methodology, Validation, Writing – original draft, Writing – review & editing. AU: Investigation, Methodology, Validation, Writing – original draft, Writing – review & editing. JL: Formal analysis, Investigation, Methodology, Writing – original draft, Writing – review & editing. IV: Conceptualization, Data curation, Formal analysis, Funding acquisition, Investigation, Methodology, Project administration, Resources, Supervision, Writing – original draft, Writing – review & editing.
