# Supplementary material for: EFhd2 co-aggregates with monomeric and filamentous tau in vitro
Source: Front Neurosci. 2024 May 3;18:1373410. doi: 10.3389/fnins.2024.1373410 (PMC11100465; doi:10.3389/fnins.2024.1373410)
Supplement: Supplementary file 1 [file Data_Sheet_1.pdf]

Supplementary Figure S1

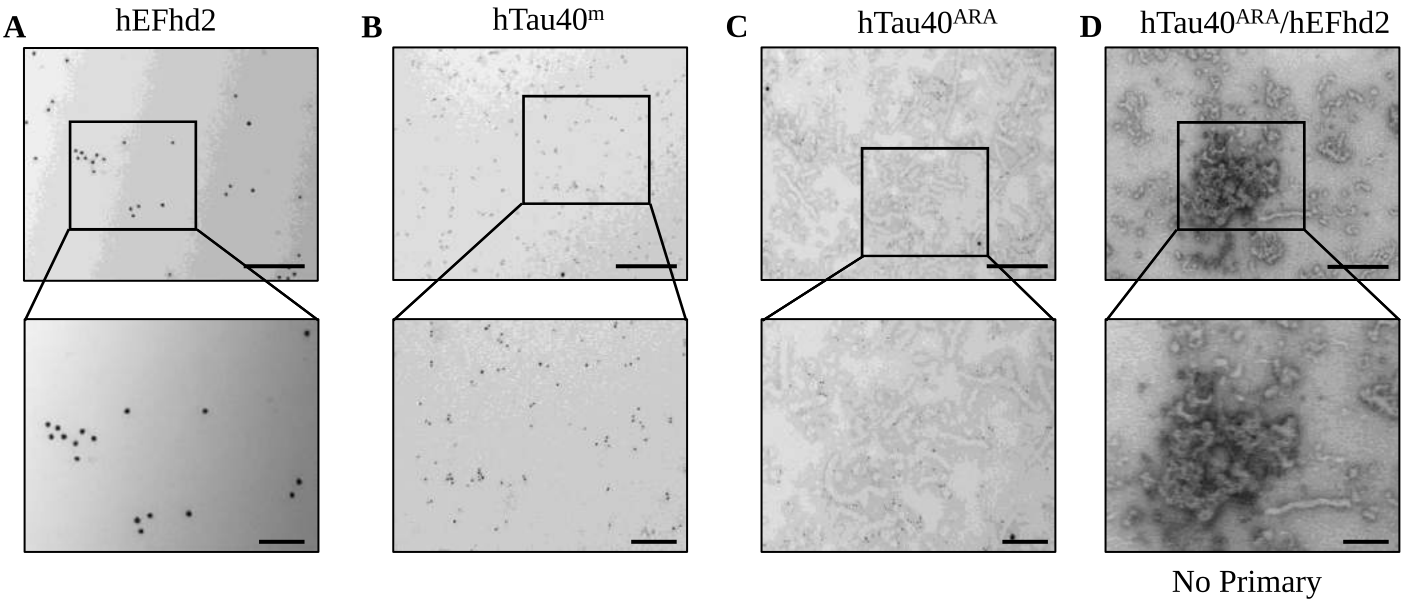


**Supplementary Figure. S1. Immunogold labeling tests the specificity of primary and secondary antibodies.** After overnight polymerization of recombinant proteins, all samples were labeled using both Tau13 antibody (IgG1 mouse antibody) and anti-EFhd2 (rabbit antibody). Then all samples were labelled with gold-conjugated secondary antibodies anti-mouse 6 nm (small gold particles) and anti-rabbit 15 nm (large particles). (**A)** Representative micrograph of immunogold labeling of hEFhd2 in the absence of ARA. Only labeling with large gold particles for hEFhd2 are detected. (**B)** Representative micrograph of immunogold labeling conducted on hTau40$^{m}$ in the absence of ARA. Only labeling with small gold particles for tau are detected. Non-specific large gold particles are scarce. (**C)** Representative micrograph of immunogold labeling conducted on hTau40 in the presence of ARA (hTau40$^{ARA}$). Only labeling of hTau40 filaments and oligomers with small gold particles are observed. (**D)** The primary antibodies for Tau13 and anti-EFhd2 were omitted to show the absence of non-specific binding of secondary antibodies to hTau40$^{ARA}$/hEFhd2 aggregates. No large or small particles were observed in the field. Data were drawn from n=3 replicates/group and 3 micrographs for each replicate.

Supplementary Figure S2

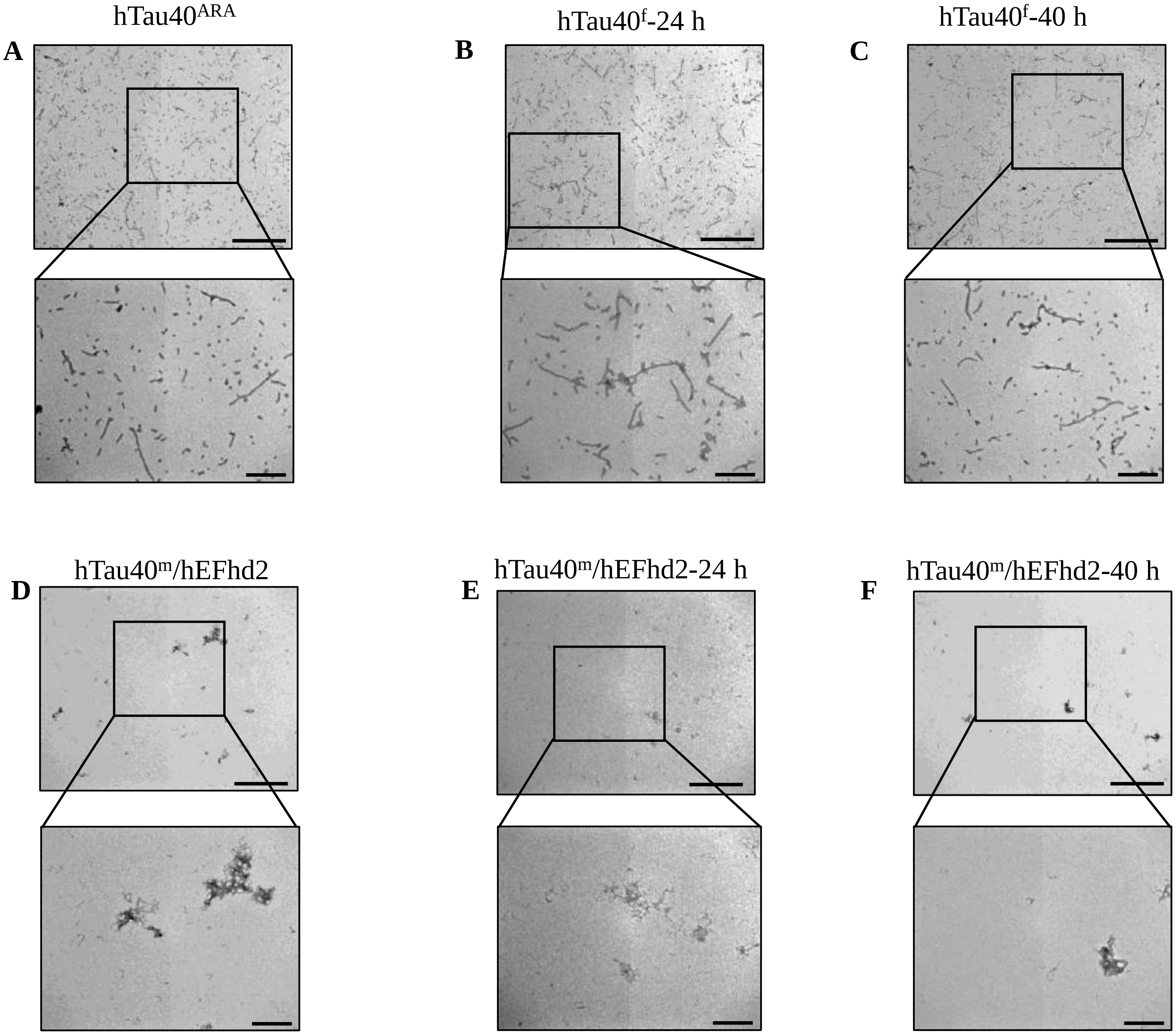


**Supplementary Figure. S2. Formation of tau filaments and hEFhd2-induced tau aggregates were not impacted by longer duration.** (**A**) Representative micrograph of hTau40$^{ARA}$ (2 μM hTau40 in the presence of ARA for 16 h); typical combination of oligomers, short and long filaments are detected. (**B**) Representative micrograph of hTau40$^{f}$ 24 h (2 μM hTau40 in the presence of ARA for 24 h before adding 2 μM hEFhd2). (**C**) Representative micrograph of hTau40$^{f}$ 40 h (adding 2 μM hTau40 in the presence of ARA for 40 h). (**D**) Representative micrograph of hTau40$^{m}$/hEFhd2 (co-incubating 2 μM of hTau40 and 2 μM hEFhd2 for 16 h in the absence of ARA). (**E**) Representative micrograph of hTau40$^{m}$/hEFhd2-24 h (co-incubating 2 μM of hTau40 and 2 μM hEFhd2 24 h before adding ARA). (**F**) Representative micrograph of hTau40$^{m}$/hEFhd2-40 h (co-incubating 2 μM of hTau40 and 2 μM hEFhd2 for 40 h in the absence of ARA). Scale bar for the top micrographs 800 nm and for the bottom micrographs 200 nm.
